# Methodology Proposal for the Management of Nursing Competencies towards a Strategic Training. A Theoretical Analysis

**DOI:** 10.3390/healthcare8020170

**Published:** 2020-06-13

**Authors:** María Begoña Sánchez-Gómez, Mercedes Novo-Muñoz, José Ángel Rodríguez-Gómez, Macarena Romero-Martín, Juan Gómez-Salgado, Gonzalo Duarte-Clíments

**Affiliations:** 1 University School of Nursing, Candelaria N.S. University Hospital, University of La Laguna, 38010 Santa Cruz de Tenerife, Spain; begonasanchez@gmail.com (M.B.S.-G.); extgduartcl@ull.edu.es (G.D.-C.); 2 Nursing Department, Faculty of Health Sciences, University of La Laguna, 38071 Tenerife, Spain; mernov@ull.es (M.N.-M.); jarogo@ull.es (J.Á.R.-G.); 3 Department of Nursing, University of Huelva, 21071 Huelva, Spain; 4 Department of Sociology, Social Work and Public Health, Faculty of Labour Sciences, University of Huelva, 21007 Huelva, Spain; 5 Safety and Health Postgraduate Program, Universidad Espíritu Santo, Guayaquil 091650, Ecuador

**Keywords:** clinical competence, competency-based education, professional competence, self-assessment, self-perception, nursing

## Abstract

Professional and academic legislation relating to nursing skills reflects conceptual and professional developments. In this sense, conceptual and methodological analyses are required to describe the concept of nursing competencies, the individual or group self-perception of competencies, to identify training needs, and to specify the nursing professional profile within the health organization. A sequential mixed methodology was proposed combining qualitative and quantitative approaches. The qualitative methodology involves the Focus Group and the Delphi technique. The quantitative methodology involves surveying and analyzing self-perception (descriptive and analytical in relation to personal and professional variables and levels of excellence). The methodology was piloted among primary care nurses. Competencies were analyzed and distributed across the training program. The combination of qualitative and quantitative methods showed that obtaining a deep insight into the nurses’ competencies would be a good process. This proposal is applicable as an approach to global nursing competencies or to a particular specialty.

## 1. Introduction

The variability of the care reality and the changes in the health needs of the population require health professionals to update their competencies by adapting them to new demands. These changes pose a challenge for nursing training, which must prepare future professionals to provide the expected effective health response. Nurse training requires nurses to strengthen, develop and broaden their own competencies, in accordance with the evolution of the context and what the citizens require [1].

Competence has been defined as the ability to use the knowledge and other skills required to successfully execute a task, develop a job, achieve a goal, or play a professional role. The concept of competence encompasses knowledge, experience, skills, personal characteristics and behaviors, beliefs, motivations, values [2]. The Harden model classifies nurse competencies into three categories: technical, intellectual and personal. In the context of health sciences, technical competencies include concepts such as practical procedures, obtaining information and communication skills. The intellectual, analytic and creative competencies include, among others, those theoretical bases and knowledge needed to carry out tasks, appropriate attitude and decision-making capabilities. Personal competencies cover the researcher’s role or functions in their own university or health system, e.g., knowledge of the system in itself and its peculiarities, their role as researcher, teacher and manager of the available resources, and as a member of multiprofessional teams. Among these competencies, the aptitude for self-criticism and self-assessment, capacity for autonomous learning, motivation, the identification of long- and short-term professional goals and their own career aspirations must also be considered [3]. Competence is a person’s ability to perform their work from a functional point of view, i.e., to be able to execute the expected behaviors in a profession by proving to be competent in a particular role and a particular work environment [4]. In addition, competencies represent a guide to clinical practice, nurse training and population-focused research that promotes interprofessional communication and the development of guidelines and protocols [5,6].

**Figure 1:**
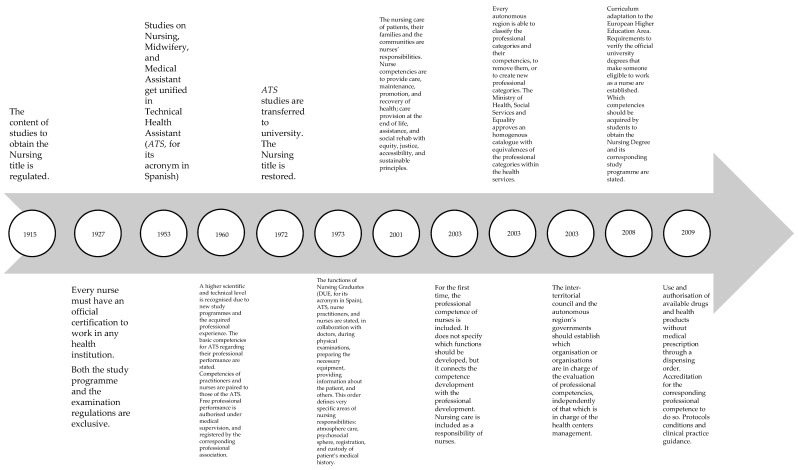
Spanish legislation on clinical and nursing training competencies.

In Spain, the concept of nursing competence has evolved in parallel with the evolution of the nursing profession, as reflected in the legislation summarized in Table 1. Initially, nursing received technical recognition. Nurses were subordinate to medical criteria, performing the assistance role during physical examinations, preparation of medical equipment and information concerning the patient. Currently, nurses are recognized as the professionals who provide care in order to maintain, promote and restore patients’ health [7,8,9,10,11,12,13,14,15] (Figure 1).

Training in competencies focuses on learning through programs designed to acquire the knowledge, skills and attitudes that students are encouraged to demonstrate at the end of their training process [1]. The European Union’s guidelines for higher education have included, among others, the organization of education according to the learning/traineeship and the introduction of competency-based education, both in undergraduate and postgraduate [16]. An education program must be drawn up based on the final expected competencies. Education based on results is an instrument of curricular planning, which shows that the product establishes the production process and not the other way around [17]. The development of the nursing curriculum has adapted the professional role to the social health needs that involve aging populations, growing dependency situations, new models of families and social roles, and technologies, making possible changes in professional performance, introducing a new competence framework adapted to social needs [18].

The development of the nursing competencies leads, naturally, to the development of the Advanced Practice Nurse (APN) [19], which maximizes the professional development of specialized competencies and nursing knowledge. The International Council of Nurses [20] defines the APN as a specialist nurse, who has achieved an expert-level of knowledge, capacity to make difficult decisions and the clinical skills needed to be a professional. The characteristics of the extended professional exercise depend on the context or the country where they are accredited to work [21]. All over the world, APNs achieve many and remarkable feats [19,20,22,23]: They improve the access to health services; reduce the waiting lists, emergency visits and the hospitalization rates and intensive care of the elderly population; upgrade the access to a proper evaluation and treatment, achieving high patient satisfaction. They make a healthier society possible and a more efficient health service [19].

Primary care is an area where nursing has expanded its role by extending its responsibilities. Home-based primary care is an effective care system for vulnerable people with health disabilities [24]. Primary care nurses are ideally positioned to cover the gap between the limits of health system coverage and access to health services [25]. The effectiveness of nurses in this area has been demonstrated. Primary care consultations attended by nurses have been equally or better valued than those attended by physicians in terms of health outcomes, patient satisfaction and cote [26,27]. Chronic patients attended by primary care nurses have fewer symptoms of depression, urinary incontinence, pressure ulcers, disuse syndrome and aggressive behaviors [28]. The time spent with each patient by primary care nurses is longer than the time spent by physicians. However, the frequency of nursing consultations is more spaced in time [27].

In Spain, the primary care reform aimed to bring health closer to citizens. This new scenario played a new role in nursing by expanding its responsibilities in areas of healthy lifestyle habits and health education promotion for individuals and families [29]. Thus, a nursing specialty dedicated to primary health care, the Family and Community Nurse, was established. These nurses teach self-care activities, motivate patients to achieve a change in attitude, design care plans for immobilized and terminal-stage patients and their caregivers, are responsible for home care and the elderly program, perform different procedures with autonomy, work on adherence to the therapeutic regimen of patients and in the self-control of chronic diseases, and lead health education, both at the health center and in the community [30].

Primary care nursing training has recently been identified as not meeting the demands of subsequent clinical practice [31]. An educational model is needed to serve as a reference for primary care nursing training programs that will bring critical thinking and decision-making together and serve as a link between practice and research [6]. To do this, it is necessary to analyze the skills required for the performance of the nursing role in primary care and the competencies included in the related training. Competency analysis allows for the design of a competency model that guides and organizes care practice, teaching and evaluation of competencies and research [31]. In addition, the evaluation of these competencies will contribute to improving the quality of nursing, adjusting the new role to current dependence needs, personalized assistance, well informed and accompanied decision-making and improving the efficiency of the health system [32,33,34].

Competence analysis studies have previously been conducted in the Nursing field. Vaughn et al. grouped advanced practice nursing competencies in rehabilitation into four domains: nurse-led interventions, promotion of health and successful living, leadership and interprofessional care [34]. In the area of public and community health, the competence analysis carried out by Campbell et al. recommended using competencies to assess clinical training, guide staff development and ongoing training, implement quality improvement projects, improve collaboration between training and practice, and develop research policies and support in the area of public health [35]. Various authors conducted a study to identify the universal nursing competencies required for registered nurses. The results described seven areas of competence: professional nursing clinical practice; professional communication and quality in nursing; determinants of health and safety in nursing; critical thinking and self-planning of professional work; new knowledge and knowledge transfer in nursing; management and coordination in nursing; and nursing research [2].

The aim of this paper is to propose a methodology for analyzing primary care nursing competencies that contribute to effective community nursing education.

## 2. Methods

In order to analyze nurses’ competencies, a sequential multimethod was proposed, combining qualitative and quantitative methodology. We suggest a combined method of qualitative (focus group and Delphi) and quantitative approach, which allows reviewing nurses’ competencies and self-assessment to identify lack in nurses’ education. It would also allow identifying the individual or group self-perception of competencies that nurses have, to generate training needs, and to specify their professional profile. Mixed methods provide in-depth knowledge of the problem, especially for those with multiple perspectives, and enables nurses to explore complex phenomena in detail [24,25]. This paper is a methodology proposal of mixed methods, which may be useful for analyzing nurses’ competencies in order to identify training needs. We propose a three steps process: (i) focus group for ideas generation about nurses’ competencies in a particular realm, (ii) Delphi technique for an agreement about the competencies identified in the previous step and the distribution along the two years of specialty training, (iii) quantitative method for self-assessing competencies among nurses, using the competencies list agreed in the previous step as a questionnaire. The proposed methodology was piloted in primary care nursing (ethical code PI-42/14).

### 2.1. Qualitative Methodology

#### 2.1.1. Focus Group

The use of the focus group is presented as a methodology to carry out research related to health, family, education, sexual behavior and other social topics [36]. A focus group is a data collection technique used in qualitative research, consisting of an in-depth interview with a group of people purposely chosen for their relationship with a particular topic, whether or not they are representative of the population [37]. Participants are summoned by the researcher to have a joint conversation in which they express their ideas, opinions or experiences about the study phenomenon [38]. Fluid dialogue offers the researcher the opportunity to explore the views of people who are considered experts on the subject, generating valuable information from their perspective [39]. The added value that characterizes the focus group is the interaction between the participants and the moderator. The exchange of opinions enriches the quality of the information obtained by allowing the members of the group to build their arguments based on the input from others and to outline their comments [40]. Another advantage of the focus group is their social component; the discussion creates an environment focused on the study topic that makes it easier for attitudes and emotions to emerge. That is, it allows contextualizing the contributions [41]. The conversation prompted by the moderator also provides information about the participants’ style of communication, common expressions, language, how to argue, etc. [39]. Additionally, the focus group is an economical and efficient data collection technique that provides the opinion of multiple participants in a short period of time [41], generating a large amount of cost-effective quality information [41]. Ideas are expressed in a synthesized way and reflect collective opinions that include the perspective of culturally diverse people [42].

In return, the focus group is more difficult to organize, as the process requires meticulous preparation in terms of the number of participants, their selection method and the moderator’s skills to generate a climate in which participants feel comfortable and the information is generated fluently [43]. In relation to the available resources, wider spaces may be needed in cases of large groups; transcriptions are more complicated, and the researchers’ interpretation is associated with a component of subjectivity [39].

Because of all the above, we consider the focus group to be an ideal method to explore a newly implemented topic such as nursing competencies from the experience of professionals. This methodology has previously been used for the analysis of nursing competencies [44,45].

Regarding the piloting in primary care nursing, 50 experts were invited to participate, and 49 out of them agreed to do so. They were invited during a seminar about primary care nursing education held by the regional health service and aimed at lecturers and mentors. As inclusion criteria, being a healthcare professional and linked to primary care nursing education was established. In order to obtain heterogeneity within the groups, professional profiles were equally distributed among the groups. The profiles of the participants were varied, with nurses (*n* = 42) and physicians (*n* = 7). According to the teaching profile, there were: nursing mentors (*n* = 5), clinical nursing tutors (*n* = 30), medical mentors (*n* = 2) and medical-clinical tutors (*n* = 7), and who did not currently hold a teaching position (*n* = 5). According to the teaching experience, 32 had teaching experience in undergraduate or postgraduate degrees, and 17 had experience in specialized training.

Participants were organized in 7 focus groups of 7 participants each. Diversity in the professional responsibility of each member was sought as a criterion of intragroup heterogeneity. Thus, in each group, there were nurses and doctors, tutors and mentors, professionals with large teaching experience and others with less experience. The focus group lasted between 90 and 135 min, depending on the internal debate.

During the focus group discussion, debate was generated following the questions “Do you think that the competencies included in primary care nursing training represent the current activity of primary care?” “Are there any activities related to primary care nursing that are not represented in competence training?” “Do you think that any concept should be expanded?” “Do you think that some competencies are not really being developed?” “What competencies do you think should be acquired in the first year of training and which in the second?”

#### 2.1.2. Delphi

The Delphi technique is particularly useful for reaching consensus among a group of experts on of a given topic so as to facilitate decision-making, solving, or prioritizing problems [46]. It is a structured process in rounds of sequential consultations with a group of experts in which consensus is progressively gained until reaching convergence of opinions [47]. The Delphi technique comprises six stages: (i) identifying a research problem; (ii) completing a literature search; (iii) developing a questionnaire of statements; (iv) conducting anonymous iterative post mail or email questionnaire rounds; (v) providing individual and/or group feedback between rounds; and (vi) summarizing the findings. This process is repeated until the degree of agreement established is reached without interaction between the participants [48].

This method offers the opportunity to have the opinion a large number of experts with geographical dispersion, that is, the group being distributed in distant locations does not pose a problem [47]. The process can be anonymous, so dominant opinions from reputable experts are avoided. The opinions of the other experts remain unknown, not being able to influence the power relations and allowing the participants to freely express themselves and autonomously change their minds [47,49]. Another advantage of this technique is that the inputs from experts are balanced, since all participants have a view in the same proportion [46]. In addition, it favors decentralization, the action of the participants is autonomous, and they share their opinion directly with the moderator, without mediation or influence, which brings together opinions that converge in agreement [50]. On the other hand, there is no unanimity in establishing consensus criteria, and ways of quantifying the agreement between participants are varied, either through statistical indices (Kappa, Cronbach’s alpha, intraclass correlation index), central trend measures, percentage of agreement [51], or content validity measures such as Lawshe’s ratio [52].

For the above reasons, the authors considered the Delphi technique as an appropriate means of seeking expert consensus on the information gathered in the previous focus groups in relation to primary care nursing competencies. Previous studies on nursing competencies analysis using this technique have been published [2,5].

In the piloting experience, the Delphi method sought to reach a consensus among a panel of experts about the suitability of the competencies identified by the focus group. Participants in the Delphi technique were asked to assess the distribution of competencies by training course.

Participants in the focus groups were invited to conform to the expert panel for the following Delphi process, 23 of which accepted to participate. The form sent by email was answered by 23 panelists in the first round (21 nurses and 2 doctors), which was resubmitted for the second round to answer the 23 questions again. Finally, in the third and last round, 21 of the initial 23 professionals participated.

**Figure 2:**
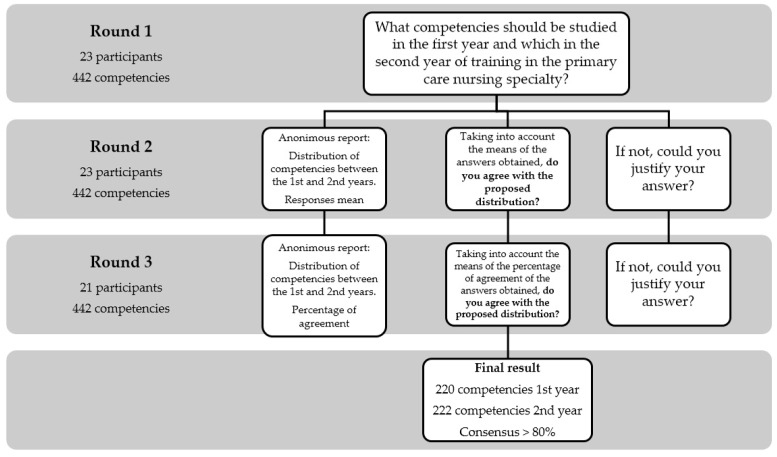
Flow chart of the Delphi research method protocol.

As shown in Figure 2, in the first round, the experts were asked the question “What competencies should be studied in the first year and which in the second year of training in the primary care nursing specialty?” Participants had 442 competencies between the two courses. To obtain a summary of each competence’s allocation to the first or second training year after the first round, the answers were gathered and the means of allocations regarding each competence were calculated. An anonymous report was prepared with the data for its distribution to the participants in the second round. Before the second round, the anonymous responses were aggregated and shared with the group to endorse and consolidate results from the first round. In the second round, participants were asked about their degree of agreement or disagreement with the allocation of competencies to the different years, considering the responses mean. The question was: “Taking into account the means of the answers obtained, do you agree with the proposed distribution? If not, could you justify your answer?” Participants responded with a dichotomous response (yes/no) for each competence. In this round, the degree of agreement of the experts was calculated with the percentage of agreement. A percentage higher than 80% was considered acceptable [51]. Once the results had been gathered and the settlement percentages calculated, a second anonymous report was drawn up and distributed to the participants in the third round. In the third round, the same procedure was followed, obtaining the final results. 

We obtained a detailed description of competencies and its final distribution during the year of nurse residency. This description indicates which areas of each competence should be addressed each year of nurse residency.

### 2.2. Quantitative Methodology

In the quantitative study, the competencies stated in the Spanish Order SAS/1729/2010, 17 June 2010, were used as a gold standard [53]. Based on these competencies and the results obtained during the previous qualitative procedure, a questionnaire was made to evaluate the self-perception of competencies of primary care nursing. The aim of this questionnaire is to identify training needs among nurses. It was as a self-administered questionnaire in which nurses could assess each competence within a Likert-type scale from 1 to 10, 1 being the lowest and 10 the highest perception of the capacity to develop each competence.

For data analysis, median as an average estimator was calculated with the responses. The data obtained were classified into cut-off points, which also identify the training needs. It is suggested to establish the cut-off points in 5, 7 and 9, so competencies could be classified as follows.

1. Answers within a range of 1–4: Additional training is required.
2. Answers within a range of 5–6: good level, but more training is required to achieve excellence.
3. Answers within a range of 7–10: excellent; it is the most challenging grade.

If we filter training needs by competencies per professional profile or function, we can get a specific and strategic analysis of the training needs. Thereby, we can diversify and optimize economic and training efforts. It is possible to personalize the training according to needs per professional profile or function in the organization.

In order to describe the results, the median and the spectrum of the obtained answers should be calculated. To evaluate the relationship between self-perception and the professional profile, it is recommended to use contrast tests of hypotheses such as the Pearson’s Chi-squared test and the Spearman rank correlation to measure the degree of association between two variables.

## 3. Results

The proposed methodology for the management of nursing competencies was piloted by analyzing primary care nurses’ competencies in the Canary Islands Health Service, Spain. These were grouped according to the experts of the focus group’s opinions about the activity of primary care nurses into six topics (Table 1):

With regard to the distribution of competencies by training years, in general, for the first year, those competencies linked to the acquisition of knowledge, assessment and recognition of critical situations or vital crises were determined, and for the second year, those that deal with psychosocial, quality and coping aspects. Table 2 differentiates those competencies that experts considered most appropriate to be studied and developed in the first or in the second year.

## 4. Discussion

In this essay, a specific study method of self-perception of competencies by nurses is presented. It is suitable for both the definition of competence needs in a specific function in the organization and the specific training needs of professionals to perform their working activity.

The legal competencies or nursing specialty legislation are the gold standards in this analysis approach, although a similar methodological approach is suitable for a global approach to nursing competencies or a particular approach in other specialties. The combination of quantitative and qualitative methods gets close to the evaluation approach in health sciences, in which the combination of diverse methodologies improves the credibility of the results. Moreover, from a strictly qualitative point of view, when the results of different proposals match and reach the same conclusion, the findings gain credibility. In this essay, the approach used combines quantitative and qualitative methods, in which results match and support each other. 

In order to conceptualize this idea, we look to various authors who share the idea that certain contributions or important information for Nursing are being devalued and distorted due to the importance given to quantitative data and measurable results [54,55,56,57]. Nurses’ performances that turn Nursing into an art form could be part of the praxis of the so-called “phronesis” or practical wisdom.

Making a self-evaluation and getting to know what is expected of oneself provides professionals with a more realistic vision as it is their perception of their own capacity. Nurses make self-evaluations, according to what is expected of them, to do and to develop. From this perspective, weaknesses during their working development can be identified and should be improved or not depending on their place/position of work, which maximizes the corporate efforts for continuous training. After all, the goal of nursing care, our “telos” or working philosophy, cannot be separated from the life development of people [58]. 

Moreover, it is important to highlight that the development of new competencies covers a broad framework. Nursing care includes the development of real competencies from real experience, practices and customs within the working environment and both the curricular and extracurricular regulated training. Thereby, the influential role of research allows confirming or denying the traditional role assumptions [59].

As mentioned throughout this essay, many authors insist on the significance and positive value that nursing competencies development have on the health system and for the citizens (patients or not) [60]. However, a disparity and variability on the development policy and its actual application by the management have been observed [61]. For this reason, it is crucial to make an effort to research this field.

From a conceptual nursing point of view, this focus approaches Abdellah’s philosophical framework [62]. She developed a system of classification of patients which was foundational for the current Diagnosis Related Group (DRGs) system. In 1960, she wrote the “21 Nursing Problems Theory”, which progressed to a second theory in 1994 referred to as “patient problems and patient outcomes”, where the problems suggested are based in relation to the Nursing services provided to determine patient needs. Her vision of Nursing takes us to a help relationship scenery where “she does what she has to do”. A nurse’s function is to do something for another person or to provide them with the necessary information to improve or to recover their self-help capacity or alleviate their suffering. This is possible through a threefold perspective: the help relationship, first of all, the patient’s needs caused by their problems and the elements of nursing care, in other words, the nurse competencies. Abdellah defends that the professional problems in Nursing, regarding the design of a professional curriculum, are caused by the lack of a unique body with scientific knowledge for Nursing, which turns into a wall for nurses in their professional advancement. The development of nurse competencies will help to improve the quality of nursing performance, visibility of nurses and their professional recognition. 

Recently, the International Family Nursing Association posted their stance on advanced practice competencies for family nursing [63,64]. In line with their related philosophical and practical vision of the nursing metaparadigm, their stance describes those competencies a family nurse should have. In the same philosophical and practical line as the competencies of a generalist nurse in family assistance previously posted [65]. The importance of research in this field takes again special emphasis as a way to give prominence to nurse performance, well defined and explained.

This methodology has some limitations as it can be subject to a kind of bias of underestimation and overvaluation, which is neutralized with the group effect. That is to say, sometimes the monitoring of a professional by a third-party can be biased: the one being monitored changes their behavior, their way of working. This is known as the Hawthorne effect [66,67]. 

## 5. Conclusions

The development of nurse competencies, particularly in advanced practice in Family and Community Nursing, has a professional and social interest and provides added value to health assistance. However, the gradual development of nursing specialties probably would require reducing competencies from a quantitative point of view, to increase them qualitatively. For this reason, further research in this field to reduce the variability and observed disparity and enable the implementation of new competencies is essential.

To sum up, it is necessary to highlight the use of methodology, particularly that of self-analysis as it allows self-assessment, evaluation of others and to design specific training strategically by professional profile or job function. In this case, the process followed by experts from the quality vision is to identify the sequence of study over a group of competencies established by law. From this, professionals decide on which point of the training development they are. This option gives professionals a chance to improve by themselves. If they become aware of their development level and what they are missing to achieve excellence, their training will be strategically planned. 

From a pluralist understanding of science, the construction of scientific models that are appropriate for certain purposes or in certain contexts may provide better resources for nursing science.

## Figures and Tables

**Table 1:** Results from the focus group regarding the analysis of primary care nurses’ competencies.

Topic	Results
Competencies that can be expanded	Emergency and catastrophe, life quality in all stages of the development
Too broad competencies	Dedication to sexual, reproductive and gender health
Competencies to be developed	Motivational interview, communication channels and tools, persuasive communication, self-concept and self-esteem, patient safety, gender-based violence
Training planning	Evolutionary training design. Community participation and family intervention should be differentiated as part of specialty care. They should be cross-sectional and must be included in other competencies: quality, research, clinical evidence, self-concept, self-esteem and the psychosocial scale in general; acquiring skills with registration and information systems, and organizational models
Knowledge management	Diagnostic accuracy and assessment of nonpharmacological therapies. The need to differentiate individual activities from collective ones is highlighted here, so as to categorize Internal Nurse Resident and Internal Medical Resident training by topics of interest and carry out joint and individual activities for each group. In addition, the continuous evaluation of competencies acquisition is considered essential
Training distinctive features	Competencies relating to coping, bereavement and the psychosocial sphere were to be treated in a specific block differentiated by each development stage. Teamwork ensures the right health care for the patient, family and community, and is one of the foundations of primary health care. General care during the adult stage should be part of the competencies to be studied during the two years of Internal Nurse Resident training. Implementing the results of the clinical practice evidence in informed and joint decision-making with the patient

**Table 2:** Results from the focus group regarding the distribution of primary care nurses’ competencies during the training.

First Year of Training	Second Year of Training
Knowledge acquisition, assessment and recognition of critical situations or vital crises	Recognize, realize, lead, intervene, design
Emergency and catastrophe care; nursing care process, confidentiality, patient safety, medication and healthcare management, personal and parental autonomy and identification of risk situations in childcare, personal autonomy, risk prevention and healthy habits in the adult stage	Training on coping, bereavement, the psychosocial sphere in general; training related to palliative care, self-help networks, social resources, addressing pregnancy, childbirth and postpartum. Assessment of health programs or education. Intervention in gender-based violence
Sex-health relationship, birth control, self-care in pregnancy, territorially based pregnant care, identification of risk factors, self-esteem, healthy lifestyle habits	Family intervention, problem management and the use of socio-family analysis methodology. Health care in situations of fragility or social health risk. Public health research, program leadership, surveillance networks, health inspection and registration, social networks and volunteering
Gender-based violence, not including intervention. Identification of risks within the family by rootlessness or isolation	
Assessment in clinical and public health. Academic assessment	Implementation of teaching programs
Scientific evidence, teamwork, process management, management of registration and information systems, patient safety, resource optimization and quality knowledge in the management of care and services at the family and community level. Identification of research needs	Team leadership, identification of gaps in information systems, promotion of the integral and continuity care, application of quality concepts and tools in the management of care and services at the family and community level. Innovation and transmission of research-described knowledge
